# Genome-Wide Expression Profile of *SOD* Gene Family in *Isatis indigotica* and the Key Role of *IiSOD2* and *IiSOD7* in Alkaline Stress

**DOI:** 10.3390/ijms26178131

**Published:** 2025-08-22

**Authors:** Lengleng Ma, Lingyang Kong, Shan Jiang, Junbai Ma, Lianqing He, Jianhao Wu, Xiaozhuang Zhang, Wei Wu, Wei Ma, Weichao Ren

**Affiliations:** College of Pharmacy, Heilongjiang University of Chinese Medicine, Harbin 150040, China; 18739366784@163.com (L.M.); hljkly970219@163.com (L.K.); 17390928032@163.com (S.J.); 15114516116@163.com (J.M.); hhelianqing@126.com (L.H.); 17515281505@163.com (J.W.); zxz999123@163.com (X.Z.); wuwei52414@163.com (W.W.)

**Keywords:** *SOD* gene family, alkaline stress, qRT-PCR, SOD enzyme activity, indole alkaloid, yeast one-hybrid key

## Abstract

Superoxide dismutase (SOD) is a key enzyme in the plant antioxidant system. It plays an essential role in plant adversity stress by scavenging excess reactive oxygen species to protect cells from oxidative damage. *Isatis indigotica*, being a mildly saline-tolerant plant, can be grown in soils containing a certain amount of saline–alkaline content. In order to reveal the *SOD* gene family members and their potential roles under saline and alkaline stress, the present study used a bioinformatics approach to identify 9 potential *IiSOD* genes in the *I. indigotica* genome. It analyzed the expression patterns of SOD family genes (*IiSODs*) in response to alkaline stress. According to the results of quantitative real-time PCR (qRT-PCR), the expression levels of the *IiSOD7* gene significantly increased within 120 h of alkaline stress treatment, while the expression level of the *IiSOD8* gene was the highest among all detected genes at 120 h of alkaline stress. The rest of the genes showed different degrees of expression. Alkaline stress showed significant and dynamic changes in the content of indigo and indirubin in leaves of *I. indigotica*. Finally, the yeast one-hybrid assay confirmed that *IiWRKY54* was able to activate the expression of *IiSOD2* and *IiSOD7*. Combined with qRT-PCR analysis, it was further hypothesized that *IiWRKY54* might enhance the alkaline tolerance of *I. indigotica* by regulating the expression of *IiSOD2* and *IiSOD7*. Taken together, this study lays the foundation for elucidating the function of the *IiSOD* gene in salinity stress tolerance of *I. indigotica* as well as promoting the genetic breeding of alkaline-tolerant varieties of *I. indigotica*.

## 1. Introduction

*Isatis indigotica* Fortune is a herbaceous biennial belonging to the genus *Isatis* of the Brassicaceae. It has a long medicinal history in Asia and Europe. The whole plant of *I. indigotica* can be used as medicine. The root of *I. indigotica* is *Isatidis* radix, which has antiviral and anti-inflammatory pharmacological effects. It is clinically used to treat influenza, hepatitis, mumps, and other diseases [1]. *Isatidis* folium has antibacterial, antiviral, antioxidant, and other pharmacological effects [2]. In addition to being medicinal, *I. indigotica* leaves or stems, through the processing of dry powder for indigo, can be used as a medicinal and a plant dye [3,4]. *I. Indigotica* is widely grown in China as a medicinal cash crop. It usually faces the threat of biotic and abiotic stress during growth and development, resulting in financial losses. Abiotic stresses mainly include saline–alkaline, drought, low temperature, and high temperature, which humans cannot control. Soil salinization is one of the key factors affecting the yield and quality of *I. indigotica*. The harmful salts in soil mainly include NaCl, Na_2_SO_4_, Na_2_CO_3_, and NaHCO_3_ [5,6].

The alkaline salt stress caused by NaHCO_3_ and Na_2_CO_3_ usually leads to the increase in pH value in the rhizosphere environment of plants, which not only destroys the physiological function of roots but also leads to the disintegration of root cells and even the destruction of root structure, thus affecting the growth and development of plants, the quality of medicinal materials and physiological and biochemical indexes [7]. The most common stress symptoms of plants under alkaline stress are chlorotic leaves and reduced plant growth to a certain extent. For example, the chlorophyll content index (CCI) of blueberry leaves in alkaline soil showed a downward trend, and the concentration of some trace elements in leaves was unbalanced, indicating that the veins of leaves were chlorotic in alkaline soil [8]. Under alkaline stress, rice causes severe damage to the growth of old leaves but has little effect on the development of young leaves [9]. Studies have shown that three genes, *GmFSD3, GmFSD4*, and *GmFSD5*, in soybean (*Glycine max* (L.) Merr.) roots were significantly induced under alkaline stress [10]. Under low temperature, high temperature, and salt stress, the two genes *HbCSD2* and *HbCSD4* in mature leaves of *Hevea brasiliensis* were significantly up-regulated to enhance stress resistance [11].

When plants are subjected to abiotic stress, they show increased respiration, decreased photosynthesis, and a large amount of ROS [12]. Excessive ROS accumulation can lead to toxic by-products of oxygen metabolism and severe cell death [13,14]. SOD is present in peroxisomes, mitochondria, chloroplasts, and cytoplasm and is the first line of defense against reactive oxygen species (ROS)-mediated damage in cells [15]. SOD protects plants from biotic and abiotic stresses by scavenging excessive reactive oxygen species produced by organisms. According to the different binding modes with metal cofactors, *SOD* genes in plants can be divided into three subfamilies: Cu/Zn-SOD, Mn-SOD, and Fe-SOD [16]. The three subfamilies of *SOD* mainly exist in higher plants, and different subtypes have different functions [17]. The *Mn-SOD* gene encodes a synthetic manganese superoxide dismutase to increase SOD activity in plants and directly scavenge reactive oxygen species to resist oxidative stress [18]. Previous studies have shown that the three subfamilies of the *SOD* gene family are present in different organelles. For example, Cu/Zn-SOD is mainly distributed in chloroplasts, cytoplasm and peroxisomes [19]. Mn-SOD is distributed in mitochondria and peroxisomes [20,21]. In contrast, Fe-SOD was mainly detected in mitochondria and chloroplasts.

In this study, we systematically analyzed the evolutionary relationships, conserved motifs, and promoter elements characterizing the *IiSOD* gene family based on the chromosome-level genome of *I. indigotica* (Size: 293.88 MB, annotated 30,323 genes encoding proteins with high confidence, diploid chromosome number 2n = 14) [22]. Based on the inhibitory effect of alkaline stress on plant growth [23], the molecular mechanism by which the *IiSOD* gene enhances plant resilience by scavenging reactive oxygen species (ROS) accumulated in excess under adverse conditions was investigated for the first time in a NaHCO_3_ alkaline stress experiment. This study, a comprehensive analysis of the *IiSOD* gene family was carried out, which provided a theoretical basis for improving the saline–alkali tolerance of *I. indigotica* cultivars.

## 2. Results

### 2.1. Identification and Physicochemical Propertiesof SOD Gene Family in I. indigotica

In this study, 8 *A. thaliana* (AtSODs) protein sequences were queried, and 9 *IiSOD* genes were identified in the *I. indigotica* genome. Based on the structural domain analysis, 3 proteins were found to have Cu/Zn-SOD domains (Pfam: 00080), 6 proteins had Fe/Mn-SOD (PF02777 and PF00081) domains. This study analyzed the protein sequence and physicochemical properties of the *IiSOD* family members. The *IiSOD* gene CDSs ranged from 462 bp to 972 bp, and the encoded IiSOD protein was 153–323 amino acids. The molecular weight of IiSOD protein ranged from 15,163.77 Da to 34,597.74 Da, and the theoretical isoelectric point (pI) ranged from 4.85 to 9.46, indicating that IiSOD protein has both acidic and alkaline proteins (Table 1).

In addition, the results of the predicted subcellular localization showed that the IiSOD protein was mainly localized in the two organelles of mitochondria and chloroplasts (Table 1). The SOD proteins of the Cu/Zn-SOD subfamily were located in the chloroplast, and the SOD proteins of the Mn-SOD subfamily were located in the mitochondria. The proteins of the same subfamily may be located in different organelles. Except that *IiSOD3* and *IiSOD7* are located in chloroplasts, other members of Fe-SOD are located in mitochondria.

According to the signal peptide prediction results of IiSOD proteins shown in Supplementary Figure S1, the SP/CS/OTHER probabilities of the IiSOD protein family are all close to 0, indicating that these proteins do not depend on the classical secretory pathway and are non-secretory proteins. The predicted transmembrane domains of the IiSOD proteins are shown in Supplementary Figure S2, and the transmembrane domains of the IiSOD protein family (Transmembrane probability ≈ 0) indicate that the IiSOD protein family does not contain transmembrane domains and does not function in a membrane-bound mode. Of these, the IiSOD1, IiSOD3, IiSOD8, and IiSOD9 proteins all exhibited significant external localization characteristics (Outside probability ≈ 1.0), with a very low internal localization probability (Inside probability ≈ 0), suggesting that these proteins are extracellular.

### 2.2. The Phylogenetic Relationship of IiSODs

**Figure 1:**
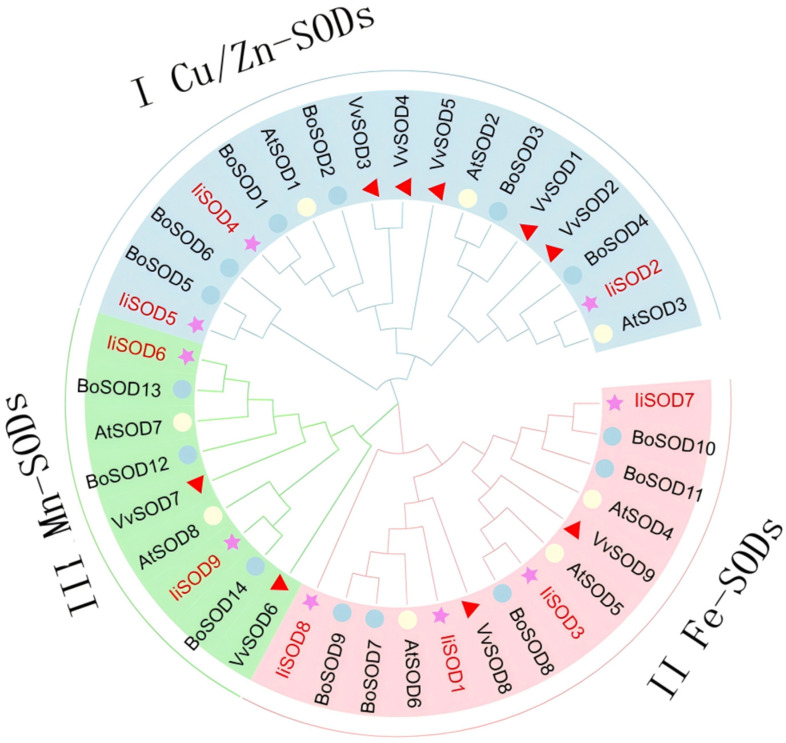
SODs phylogenetic tree of *I. indigotica*, *A. thaliana*, *B. oleracea*, and *V. vinifera*, 40 SOD proteins were constructed using the neighbor-joining (NJ) method. The SOD protein is divided into I Cu/Zn-SODs, II Fe-SODs, and III Mn-SODs. The proteins of the *I. indigotica* are labeled with red, and various colors and shapes distinguish different varieties of SODs.

Phylogenetic trees are widely used to show the evolutionary relationships of gene families. Phylogenetic analysis of SOD protein sequences of *I. indigotic (nine*), *A. thaliana* (eight), *Brassica oleracea* (fourteen), and *Vitis vinifera* (nine) was used (Supplementary Table S1). Based on domain analysis and the phylogenetic tree, the *SOD* genes of all species were clustered into three major clades: Cu/Zn-SODs (I), Fe-SODs (II), and Mn-SODs (III), represented by light blue, pink, and light green, respectively (Figure 1). SOD protein sequences clustered in the same branch are more likely to exhibit functional similarity. The Cu/Zn-SOD group contained 17 SOD members (3 *liSODs*, 3 *AtSODs*, 6 *BoSODs*, and 5 *VvSODs*), the Fe-SOD included 14 members (4 *liSODs*, 3 *AtSODs*, 5 *BoSODs*, and 2 *VvSODs*), and the Mn-SOD group had 9 members (2 *liSODs*, 2 *AtSODs*, 3 *BoSODs*, and 2 *VvSODs*). The *I. indigotica*, *A. thaliana*, and *B. oleracea* belong to the cruciferous family, and the SOD family members are closest to the *AtSOD* and *BoSOD* gene family members in the phylogenetic tree. The results showed that the *IiSOD*, *AtSOD*, and *BoSOD* families were most closely related during evolution. Interestingly, the Cu/Zn-SOD and Fe-SOD groups of each species contained more SOD compared to the Mn-SOD group. In the Fe-SOD group, *IiSOD8*’s similarity to *A. thaliana* Fe-SOD (50%) was significantly higher than its similarity to *A. thaliana* Mn-SOD (20%).

### 2.3. Chromosomal and Collinearity Analysis of IiSODs Gene

**Figure 2:**
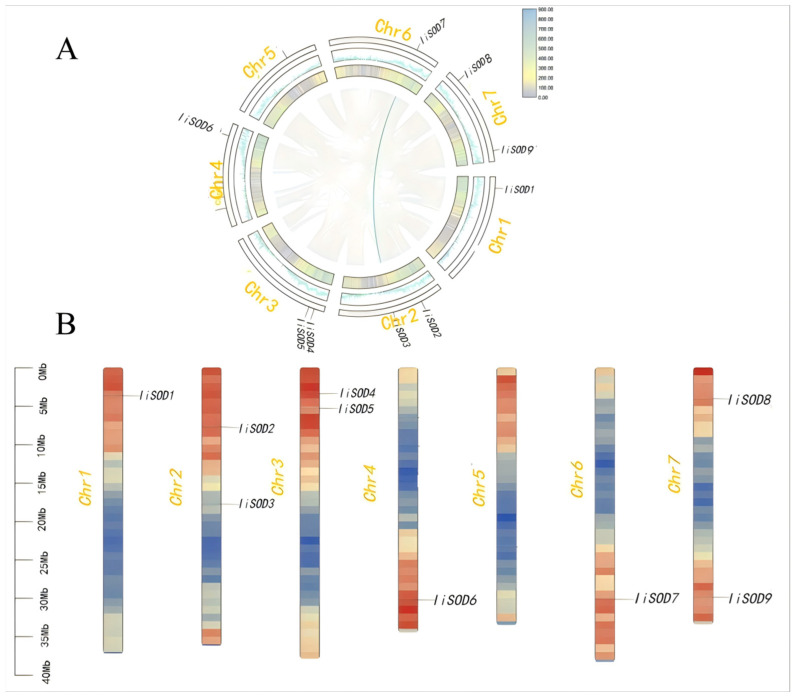
Collinearity analysis and chromosomal localization of *IiSODs* gene. (**A**) Interspecific collinearity analysis of the *IiSODs* gene. The gray background shows all the same linear segments in the genome of *I. indigotica*. The blue line shows the identical linear *IiSOD* gene pairs. (**B**) The number of chromosomes of the *IiSODs* gene are shown on the left side of each chromosome. The respective genes were marked on the right side of the chromosome.

According to the genome annotation file of *I. indigotica*, we analyzed the relative position of 9 *IiSODs* on chromosomes. The results confirmed that 9 *IiSOD* genes were distributed on seven different chromosomes. According to the chromosome distribution of *A. thaliana* and the widely accepted naming system, the *IiSOD* candidate genes were named *IiSOD1-9.* Among them, Chromosomes 2, 4, and 7 each have two genes distributed, and strangely, there is no gene distribution on chromosome 5. The results showed that the positional distribution of *IiSOD* genes was irregular and, in addition, no tandem duplication events were found (Figure 2B). Collinearity analysis revealed that a segmental duplication occurred in one gene pair (*IiSOD3* and *IiSOD7*) in the *IiSOD* genes (Figure 2A), and they both belonged to the Fe-SOD subfamily. *IiSOD3* and *IiSOD7* may have undergone subfunctionalization, neofunctionalization or functional redundancy.

### 2.4. Conserved Motifs and Domains of IiSOD Proteins

**Figure 3:**
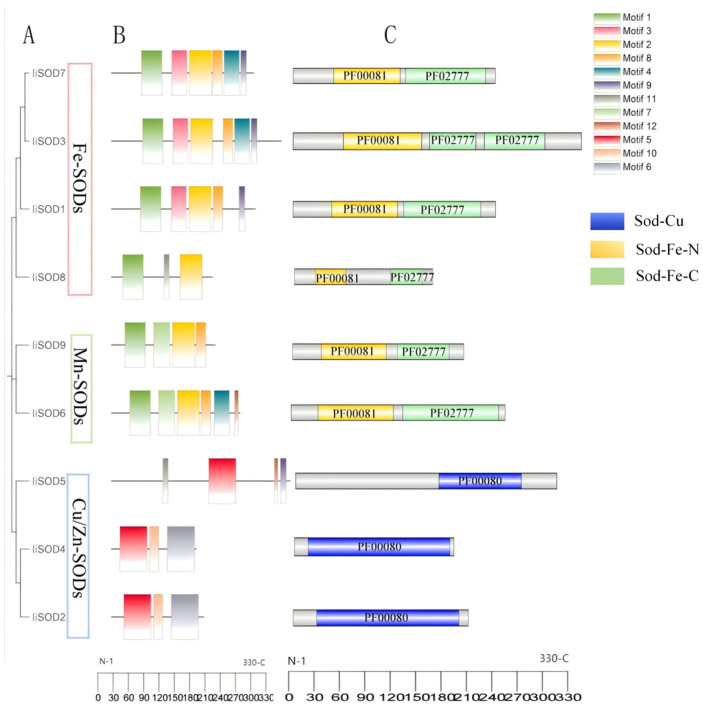
Phylogenetic tree, conserved motifs, and domain analysis of IiSOD. (**A**) Neighbor-joining tree of IiSODs protein. (**B**) IiSOD conserved motif analysis. (**C**) Conserved structure domains of IiSOD. The blue, yellow, and green rectangles indicate the SOD_Cu structural domain (PF00080), SOD_Fe_N structural domain (PF00081), and SOD_Fe_C structural domain (PF02777), respectively.

The nine liSODs were divided into two groups (Figure 3A): one group consisted of three Cu/Zn-SODs, while the other group included six proteins: four Fe-SODs and two Mn-SODs. We further analyzed the conserved motifs of *IiSOD*, and showed that each subfamily of *IiSOD* has a unique motif composition (Figure 3B). These conserved motifs may have specific roles in their respective subgroups. Among them, motif 5 is related to the Cu/Zn-SOD domain (PF00080) and is only present in members of the Cu/Zn-SOD subfamily. Conserved motifs 1 and 2 are present in both Fe-SOD and Mn-SOD families, and motifs 5, 6, and 10 are contained in the Cu/Zn-SOD family. Each subfamily contains the same conserved motif. Analysis of the conserved domains showed that both IiFe-SODs and IiMn-SODs contained SOD_Fe_N (PF00081) and SOD_Fe_C (PF02777) domains among the nine liSODs, while IiCu/Zn-SODs contained only Sod_Cu (PF00080) domains (Figure 3C).

### 2.5. Cis-Regulatory Elements in the Promoter Region of IiSODs Gene

**Figure 4:**
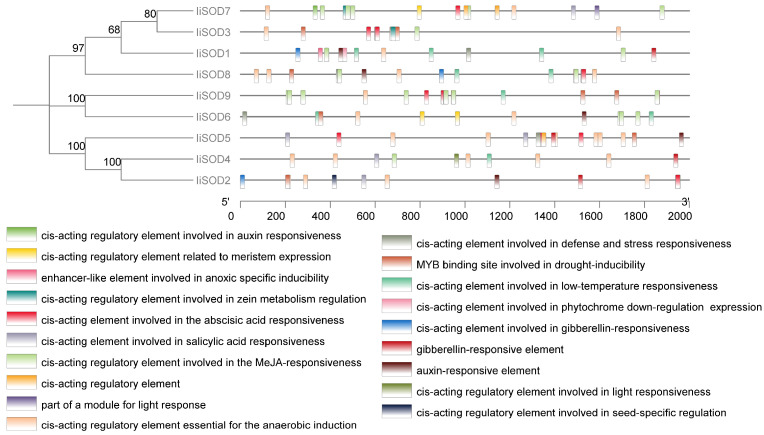
The cis-regulatory elements in the promoter region of the *IiSOD* gene family. Different colors in the *IiSODs* promoter region indicate cis-elements with various functions.

To predict the functions and regulatory roles of *IiSOD* genes, a region of 2000 bp upstream of the transcriptional activation sites of *IiSOD* genes was extracted using the plantCARE database, and cis-regulatory elements in the promoter regions of *IiSODs* were examined (Figure 4). The results showed that the promoter region of *IiSODs* contained abundant cis-regulatory elements, including the binding sites of MYB, and MYC, (Figure 4). The hormone-responsive cis-elements were mainly abscisic acid (ABRE), methyl jasmonate (MeJA), salicylic acid (SA), growth hormone, and gibberellin (GA). The promoters of *IiSODs* commonly contain MYC binding sites (MYC) and anaerobic-inducible elements (ARE), suggesting that the *IiSOD* gene may be directly regulated by bHLH-like MYC transcription factors, which can respond rapidly to jasmonic acid, saline drought, and oxidative stresses, and that the *IiSOD* gene can be activated by hypoxic signals to maintain ROS homeostasis, and that it has a combination of both antioxidant and hypoxia tolerance functions. In summary, *IiSOD* genes have different expression levels and functions under different environmental conditions and are involved in responding to various stresses.

### 2.6. IiSOD Gene Expression Profile Under Alkaline Stress

**Figure 5:**
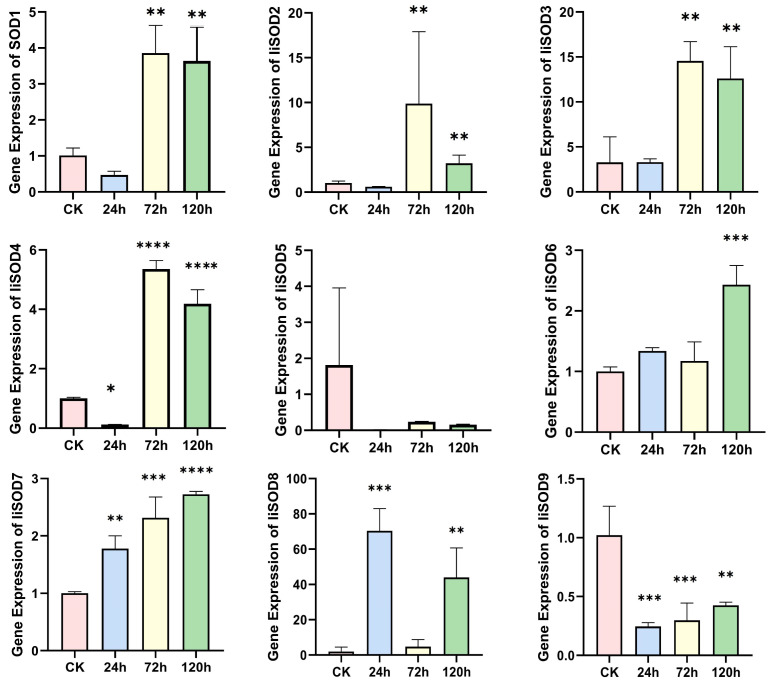
Expression level of the *IiSOD* gene under alkaline stress. Data are means (mean ± SD) of three replicates. The significance of *IiSOD* gene expression level was analyzed by one-way analysis of variance (ANOVA) and Tukey’s multiple comparison test (* *p* < 0.05, ** *p* < 0.01, and *** *p* < 0.001, **** *p* < 0.0001).

Under alkaline stress, the nine *IiSOD* genes showed significant differences (Figure 5). The expression of *IiSOD7* was low in the CK group. It significantly increased in the treatment group, suggesting its expression was time-dependent under alkaline stress. The expression levels of *IiSOD1*, *IiSOD2*, *IiSOD3*, and *IiSOD4* remained almost unchanged at 24 h of stress, reaching their peak after 72 h. Subsequently, the expression level decreased at 120 h of stress, showing a typical stress response pattern of “delay-peak-attenuation”. *IiSOD6* was up-regulated after 24 h of stress and reached a peak at 120 h of stress. It is worth noting that *IiSOD8* exhibits a dynamic change in increase-decrease-increase under alkaline stress, with expression reaching a peak at 24 h, rapidly declining at 72 h, and rising slightly again at 120 h. Furthermore, its expression level is the highest among all detected genes, indicating that *IiSOD8* exhibits temporal fluctuations in response to alkaline stress. On the contrary, the *IiSOD5* gene was completely silenced at 24 h under alkaline stress, indicating that it did not respond to early stress or was specifically inhibited. Although *IiSOD9* showed a slow upward trend with stress time, the overall expression level was always the lowest.

### 2.7. Determination of Phenotype and SOD Activity of I. indigotica Under Alkaline Stress

**Figure 6:**
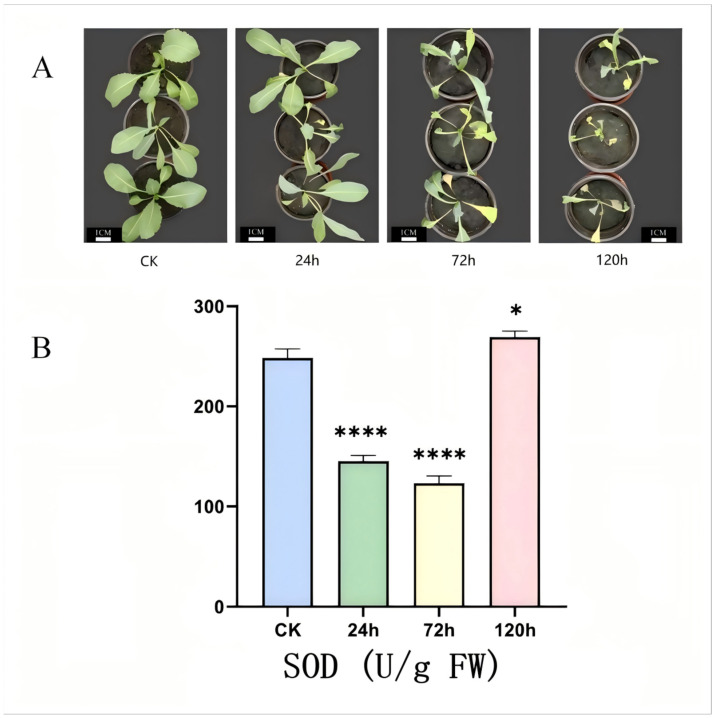
Effects of alkaline stress on the phenotype and SOD enzyme activity of *I. indigotica*. (**A**) Phenotype of *I. indigotica*. The plant height of *I. indigotica* decreased, leaves wilted, and growth was reduced with increasing treatment time. (**B**) SOD activity. Data are means (mean ± SD) of three replicates. Significance of SOD enzyme activity was analyzed using one-way ANOVA followed by Tukey’s multiple comparisons test (*: *p* < 0.05, ****: *p* < 0.0001).

After alkaline stress treatment, the phenotype of *I. indigotica* showed significant differences in leaf color and growth status, such as leaf chlorosis, yellowing and decreased growth. With the extension of alkaline stress treatment time, the effect of alkaline stress on plant phenotype is becoming increasingly apparent. Especially in the final sampling stage, the phenotypic differences are pronounced. The CK group showed higher plant height and healthier leaves. In contrast, the plant height of the 120 h alkaline stress treatment group decreased, the leaves wilted and the growth amount was seriously reduced (Figure 6A). Under the same concentration of NaCl salt stress, the effect on plant phenotype was relatively small. The leaves remained evergreen under stress for 72 h, but the leaves showed severe chlorosis and wilting, and yellowing at 120 h [24].

With the prolongation of alkaline stress time, the activity of SOD decreased first and then increased, indicating that the oxidative stress induced by alkaline stress activated the antioxidant defense system. Our previous studies have found that the level of reactive oxygen species in *I. indigotica* increased sharply under stress, which led to oxidative damage of cell structure in plants and usually started the antioxidant enzyme system to resist the increase in reactive oxygen species content [24]. As the first line of defense of the antioxidant enzyme system, SOD directly scavenges oxygen free radicals in plants and reduces the accumulation of reactive oxygen species. Therefore, the activity of the SOD enzyme in *I. indigotica* tissue samples is an important indicator to observe the antioxidant capacity of *I. indigotica*. The SOD activity of *I. indigotica* showed an increasing trend under alkaline stress treatment by the superoxide dismutase kit (Figure 6B). The SOD activity of *I.indigotica* plants decreased first and then increased under alkaline therapy at different times. Compared with the control group, the enzyme activity of SOD decreased under alkaline treatment for 24 h and 72 h, and the enzyme activity of SOD increased to the maximum value with the extension of alkaline treatment to 120 h. Therefore, it can be seen that in a specific concentration range, *I. indigotica* can resist the damage caused by reactive oxygen species under alkaline stress by increasing the activity of the SOD enzyme in the antioxidant system. The SOD activity was calculated using the following formula:

$$SOD\;activity\;(U/g)=\frac{A1\times V1\times V2\times D}{(1-A1)\times W\times V3}$$

A1: inhibition percentage; V1: total volume of the reaction system; V2: total volume of crude enzyme solution; D: dilution factor of crude enzyme solution; W: sample mass; V3: volume of crude enzyme solution added to the reaction system.

### 2.8. Content of Indole Alkaloids in Leaves of I. indigotica Under Alkaline Stress

**Figure 7:**
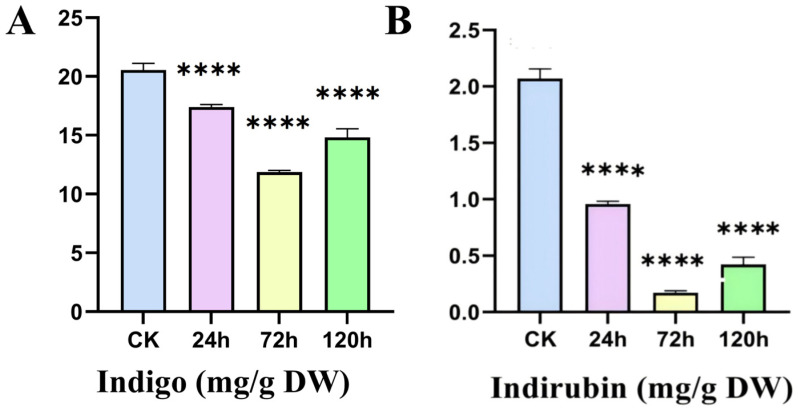
Effect of alkaline stress on indigo and indirubin content in leaves of *I. indigotica*. (**A**) Indigo content; (**B**) indirubin content. Data are means (mean ± SD) of three replicates. One-way ANOVA followed by Tukey’s multiple comparisons test was used for the significance of chemical content in leaves of *I. indigotica* under alkaline stress (****: *p* < 0.0001).

The content of indigo and indirubin in leaves of *I. indigotica* under alkaline stress showed a tendency of decreasing and then increasing with the prolongation of stress time (Figure 7). From the figure, it can be seen that the indigo content in *I. indigotica* leaves continued to decrease at 24 h and 72 h of alkaline stress, and the indigo content slightly recovered at 120 h, but it was still lower than that of CK (Figure 7A). The trend of indirubin was similar to that of indigo, which also showed a decrease and then an increase, but the decrease was more significant: at 24 h of alkaline stress, the content of indirubin decreased by about 50%, indicating that its sensitivity to alkaline stress was higher than that of indigo (Figure 7B). Supplementary Figure S3 shows the HPLC of indigo and indirubin under alkaline stress. The peak area is shown in Supplementary Table S4.

### 2.9. IiWRKY54 Binds to IiSOD2 and IiSOD7 Promoter Genes

**Figure 8:**
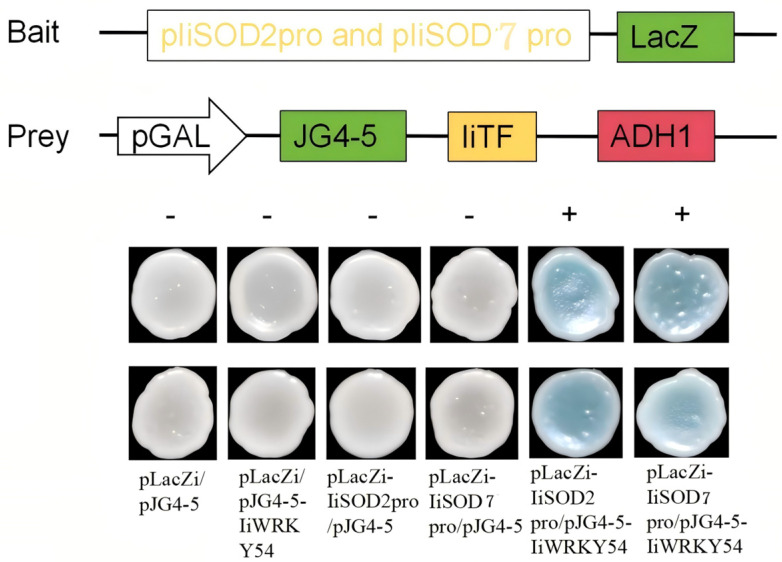
Yeast one-hybrid screening of target genes that bind to *IiWRKY54*. The colony color of pLacZi-*IiSOD2pro* or pLacZi-*IiSOD7pro* and pJG4-5-*IiWRKY54* became blue, indicating that the *IiWRKY54* transcription factor interacts with *IiSOD2* or *IiSOD7* genes.

According to the transcription factor of the alkaline-tolerant gene *AtWRKY45*, the phylogenetic tree was constructed with *IiWRKY* to screen *IiWRKY54* for a close genetic relationship. Through yeast one-hybrid experiments, the vector pJG4-5-*IiWRKY54* transcription factor was co-transformed into the Saccharomyces cerevisiae strain with the vector pLacZi-*IiSOD2* or pLacZi-*IiSOD7* promoter. Under the appropriate temperature conditions, the colony color changed to blue (Figure 8). This indicates that the *IiWRKY54* transcription factor can activate the transcription of the *IiSOD2* or *IiSOD7* gene. *IiWRKY54* can regulate the transcription of the downstream *IiSOD2* or *IiSOD7* gene promoter, thereby improving the stress response of *I. indigotica* to alkaline stress.

## 3. Discussion

Excessive accumulation of ROS under adversity stress in *I. indigotica*, an important medicinal and economic crop, significantly reduces biomass and affects the synthesis of secondary metabolites. SOD, as the first line of defense against oxidative stress in plants, plays a key role in maintaining ROS homeostasis by catalyzing the disproportionation reaction of superoxide anion (O_2_^−^). By systematically analyzing the functional regulatory mechanism of the *IiSOD* gene family under adversity stress, we can provide a theoretical basis for cultivating high-quality and stress-resistant varieties. The *SOD* gene family has been comprehensively studied in a variety of plants, such as *Liriodendron chinense* [25], *Triticum turgidum* [26], and *Cassava* [27]. This paper is based on the genome file of *I. indigotica*; 9 *IiSOD* genes were successfully identified in *I. indigotica* by bioinformatics methods, which was similar to the number of *SOD* genes reported in most plants. For example, there are 8 *SOD* genes in *A. thaliana*, 8 *SOD* genes in *Liriodendron chinense* [25], 14 *SOD* genes in *Triticum turgidum* [26], 8 *SOD* genes in Cassava [27], and 22 *SOD* genes in *Ophiopogon japonicus* [28]. Nine *SOD* genes were identified in *Rosa roxburghii* Tratt [29].

Physicochemical properties analysis showed that the *IiSOD* family was mainly in mitochondria and chloroplasts. In the Fe-SOD subfamily, except for *IiSOD3* and *IiSOD7*, which are located in chloroplasts, the remaining members, *IiSOD1* and *IiSOD8*, are distributed in mitochondria (Table 1) [30]. A phylogenetic tree was constructed using the *SOD* sequences of and *I. indigotica*, *A. thaliana*, *B. oleracea*, and *V. vinifera*. *IiSOD* (9), *A. thaliana* (8), *B. oleracea* (14), and *V. vinifera* (9) proteins were divided into three subfamilies. The *I. indigotica*, *A. thaliana*, and *B. oleracea* belong to the cruciferous family, and the SOD family members are closest to the *AtSOD* and *BoSOD* gene family members in the phylogenetic tree. Phylogenetic analysis showed that the members of the *IiSOD* gene family could be divided into three subfamilies: Fe-SOD, Mn-SOD, and Cu/Zn-SOD according to their sequence characteristics, and the members of the same subfamily were usually distributed in the same cell compartment, which was consistent with the report of SOD gene clustering and subcellular localization [31].

The conserved motifs of *IiSOD* are consistent across subfamilies, suggesting that gene functions may be similar within the same subfamily. In addition, conserved motif analysis further revealed significant differences between different subfamilies, such as the differentiation of Fe-SOD and Cu/Zn-SOD subfamilies in functional domains (Figure 3B) [32]. IiCu/Zn-SODs proteins contain the conserved SOD_Cu domain (Pfam: 00080), both IiFe-SOD and IiMn-SOD possess the Sod_Fe_N (Pfam: 00081) and Sod_Fe_C (Pfam: 02777) domains. These proteins are classified into three subfamilies, consistent with previous studies [33]. Fe-SODs and Mn-SODs from different plants come together and are separated by high guide values, suggesting that they may have originated from a common ancestral gene [34].

PlantCARE analysis showed that there were a large number of cis-acting elements in the promoter region of the *IiSOD* gene family in response to plant hormones (Figure 4), which was consistent with the fact that the *SOD* gene was regulated by ABA and MeJA in plants [35,36]. The promoter region of the *IiSOD* gene has a large number of MYB binding sites. Analysis of cis-acting elements in *I. indigotica* promoters showed different regulatory patterns of indigo growth and response to stress [37].

The qRT-PCR results showed that the *IiSOD7* gene responded positively to alkaline stress, whereas the *IiSOD8* gene had the highest expression levels under alkaline stress. This suggests that both the *IiSOD7* and *IiSOD8* genes play key roles in the response of *I. indigotica* to alkaline stress. The results of qRT-PCR combined with phylogenetic analysis showed that the *IiSOD* gene in response to alkaline stress was a member of the Cu/Zn-SOD and Fe-SOD subfamily. It could be inferred that the SOD protein types that played a role in scavenging reactive oxygen species in the leaves of *I. indigotica* under alkaline stress were Cu/Zn-SODs and Fe-SODs. In summary, qRT-PCR results showed that the expression patterns of *IiSOD* genes in *I. indigotica* under alkaline stress treatment were differentially expressed, indicating that these genes were specific in response to environmental stress under alkaline stress treatment.

Indigo and indirubin are secondary metabolites of *I. indigotica* formed by oxidative polymerization of indole precursor compounds (e.g., indoxyl, isatin) under adverse conditions [38]. HPLC analyses showed that the contents of indigo and indirubin showed a dynamic trend of decreasing and then increasing under alkaline stress, indicating that the response of *I. indigotica* to alkaline stress has a stage-regulated characteristic. In the early stage of stress, the osmotic and ionic stress triggered by the high pH environment prompted the plants to preferentially allocate carbon–nitrogen resources to the synthesis of osmotic-regulating substances and the maintenance of ionic homeostasis, resulting in the inhibition of secondary metabolic pathways and a decrease in the content of indigo and indirubin [39,40]. As the stress persisted, cells initiated secondary defense responses by activating the Ca^2+^-ROS-MAPK signaling cascade [41], at which time indigo and indirubin were synthesized in large quantities and their contents were significantly reduced due to their ROS scavenging ability [42,43].

It is worth noting that the indigo content of CK group leaves was as high as 20 mg/g at 0 h; after 72 h of alkaline stress, this value decreased to a minimum of 12 mg/g, a decrease of 40%. The content of indirubin also showed a synchronous decline, with both showing similar trends. The above results indicate that an alkaline environment significantly inhibits the accumulation of indigo and indirubin in *I. indigotica* leaves. This result is consistent with the drought and salt stress data detected by UPLC: in this study, the content of indigo, indirubin, tryptanthrin, and syringic acid in *I. indigotica* was significantly reduced [44].

According to the results of the Y1H experiment, the yeast colonies co-transformed with pLacZi-*IiSOD2* or pLacZi-*IiSOD7* and pJG4-5-*IiWRKY54* showed blue, indicating that *IiWRKY54* can specifically bind to the promoters of *IiSOD2* and *IiSOD7* and significantly activate their transcription. Because *IiSOD2* and *IiSOD7* are key genes in plant response to alkaline stress, and the overexpression of *IiSOD7* can enhance the resistance of *I. indigotica*, *IiWRKY54* may positively regulate the expression of *IiSOD2* or *IiSOD7*, thereby improving the tolerance of *I. indigotica* to alkaline stress.

## 4. Materials and Methods

### 4.1. Plant Material and Treatment Sampling

The seeds of *I. indigotica* are from the Medicinal Botanical Garden of Heilongjiang University of Chinese Medicine. Firstly, the seeds of *I. indigotica* are screened, and the seeds with whole seeds and bright seed coats are selected for sowing. The seeds of *I. indigotica* were sown in a seedling tray containing vermiculite and nutrient soil (1:4), and one seed was sown in each hole. When the plant grows to 3–4 leaflets, it is transferred to a pot (15 cm high, 15.3 cm in diameter), and one plant is planted in each pot. When *I. indigotica* grows for about 70 days and reaches 5–6 leaves, it is ready for treatment.

Based on the fact that soils in parts of Heilongjiang, China, are characterized by typical soda salinity, with a salt composition dominated by sodium carbonate (Na_2_CO_3_) and sodium bicarbonate (NaHCO_3_), and with measured HCO_3_^−^ concentrations ranging from 100 to 250 mmol/L (pH 8.0–9.2) [45,46,47]. To simulate the effect of Heilongjiang saline soil on the growth status of *I. indigotica*. A 200 mmol/L NaHCO_3_ alkaline solution (pH ≈ 8.2) was prepared to stress *I. indigotica* [48]. The experiment was set up for four stress treatment times, and three plants with uniform growth were selected as biological replicates for each stress group. The root soil of uniformly growing *I. indigotica* plants was watered with 200 mL of NaHCO_3_ alkaline solution. The 0 h (CK) group was watered with the same volume of sterile water for the same duration of stress. All plants were sampled at 24 h, 72 h, and 120 h after the start of treatment under the same cultivation and management conditions. The samples were frozen in liquid nitrogen immediately after collection and stored in a refrigerator at −80 °C.

### 4.2. Identification and Physicochemical Properties of IiSOD Gene in I. indigotica

The *I. indigotica* genome was characterized by two methods, protein blast (BlastP) and Hidden Markov Model (HMM) [49]. *I. indigotica* genome and annotation files were obtained from the Figshare database (Available online: https://figshare.com/) (accessed on 4 January 2025) [2,22]. The amino acid sequences of the eight AtSODs were used as the query sequences, and the E-value was set to 1 × 10^−5^ to search for potential *IiSOD* candidate genes. The amino acid sequences of the *AtSODs* were obtained from the *A. thaliana* genome database TAIR (Available online: http://www.arabidopsis.org/ (accessed on 10 January 2025)). The HMM spectra of Cu/Zn-SOD (PF00080) and Fe/Mn-SOD (PF02777 and PF00081) were downloaded from the InterPro database. Then, the IiSOD protein database was scanned using TBtools v2.210 software’s HMM search [50].

The MEME website (Available online: https://meme-suite.org/meme/db/motifs (accessed on 16 January 2025)) parameter is set to 12, and the rest of the default parameter values are selected to identify the conserved motifs in the IiSOD protein [51]. TBtools v2.210 software was used to visualize the conserved motifs of the *IiSOD* gene. The physical and chemical properties of IiSOD protein, such as molecular weight and isoelectric point (pI), were calculated using the ExPASy online website (Available online: https://web.expasy.org/compute_pi/ (accessed on 20 January 2025)) [52]. In addition, WoLF PSORT (Available online: https://www.genscript.com/tools/wolf-psort (accessed on 2 March 2025)) was used to predict the subcellular localization of IiSOD protein [53]. Signal peptides of IiSODs protein were predicted by the SignalP 5.0 website (Available online: https://services.healthtech.dtu.dk/services/SignalP-5.0/ (accessed on 8 March 2025)). The transmembrane structural domains of IiSOD proteins were predicted by the online TMHMM-2.0 website (Available online: https://services.healthtech.dtu.dk/services/TMHMM-2.0/ (accessed on 16 March 2025)).

### 4.3. Phylogenetic and Collinearity Analysis of IiSOD Protein

To observe the evolutionary relationship of the *IiSOD* gene family, the phylogenetic tree of *I. indigotica* and *A. thaliana* protein sequences was constructed. MEGA 11 software was used for sequence alignment, and IiSOD protein sequences were selected by the neighbor-joining (NJ) algorithm to build a rootless phylogenetic tree. The phylogenetic relationship between *I. indigotica* and *A. thaliana SOD* genes was studied. Parameter settings include the model being p-distance, the missing data method being partial deletion, the proportion being 50%, Bootstrap is set to 1000, and other parameters being default parameters [54]. The phylogenetic tree was beautified using the Evolview website (Available online: https://www.evolgenius.info/evolview/ (accessed on 22 March 2025)) online tool. The IiSOD protein position was extracted from the GFF3 annotation file of the genome of *I. indigotica*, and then the intraspecific collinear visualization was performed using the Circos of TBtools v2.210 v2.0 software.

### 4.4. Analysis of Cis-Acting Elements in IiSOD Promoter

Analysis of cis-elements in the *IiSOD* promoter helps understand information on gene expression regulation. TBtools v2.210 software extracted the gene structure information from the GFF3 gene annotation file of *I. indigotica*, and the sequence 2000 bp upstream of the *IiSOD* gene was further extracted [55]. The PlantCARE website (Available online: http://bioinformatics.psb.ugent.be/webtools/plantcare/html/ (accessed on 28 March 2025)) was used to predict cis-acting elements, and the expected results were screened and simplified [56]. Finally, the results are visualized by TBtools v2.210 software.

### 4.5. RNA Extraction and qRT-PCR Analysis of I. Indigotica

Total RNA was extracted from *I. indigotica* plants under different temporal alkaline stress treatments using a total RNA extraction kit (DP441; Xinjing, Hangzhou, China). To minimize individual differences, only one fully expanded leaf (counting from top to bottom, the 3rd–4th leaves) was taken from each *I. indigotica* plant in each treatment group and mixed in equal amounts for RNA extraction. The concentration of RNA was then determined using an ultra-micro UV spectrophotometer (Eppendorf, Hamburg, Germany). Subsequently, the extracted *I. indigotica* RNA was reversely transcribed into *I. indigotica* cDNA using the Vazyme reverse transcriptase kit (Novozan, Nanjing, China).

The qRT-PCR detected the expression pattern of the *IiSOD* gene under alkaline stress. The qPCR primers were designed using the Primer 3 website [57], and the EF1-α gene was used as a reference gene. The gene sequences and primer sequences used are detailed in Supplementary Table S2. The qRT-PCR analysis was performed using a fluorescence quantitative PCR instrument (Agilent Technologies, Santa Clara, CA, USA). The specific procedures were as follows: 95 °C 3 min, followed by 40 cycles of primer annealing/extension, 95 °C 30 s, 60 °C 10 s and 95 °C 30 s, and finally one cycle of 95 °C 30 s, 65 °C 30 s and 95 °C 30 s. Three biological replicates were performed for each sample, and three technical replicates were used for each replicate. The relative expression of *IiSOD* genes was calculated using the 2^−ΔΔCt^ method [58]. Expression levels of *IiSOD* genes were plotted using GraphPad. Prism. 8 software.

### 4.6. Phenotypes and Superoxide Dismutase Activity in Alkaline Stress

*I.indigotica* was stressed using an alkaline solution of 200 mmol/L NaHCO_3_. In each treatment group, 200 mL of NaHCO_3_ solution was poured on the *I. indigotica* root soil, while the same volume of sterile water was used for the blank group. Changes in phenology of *I. indigotica* plants regarding plant height, color, volume, degree of wilting, and fresh weight were recorded under the same cultivation and management conditions at 0 h (CK), 24 h, 72 h, and 120 h alkaline treatments. Photographs were taken in each treatment group at the time of sampling. The *I. indigotica* were frozen in liquid nitrogen and stored at −80 °C.

SOD enzyme activity assay kit (G0101W, Suzhou Greus Biotechnology Co., Ltd., Suzhou, China) was used to determine the activity of SOD enzyme in *I. indigotica* tissue samples by the WST-8 method. Weighed 0.1 g of *I. indigotica* tissue under different alkaline stress treatments, homogenized with the extract provided by the 1 mL kit under ice bath conditions, homogenized and centrifuged at 4 °C × 12,000 rpm for 10 min, and the supernatant was taken as the test solution. According to the kit’s instructions, the required reagent was added to a 96-well detachable microtiter plate (12 wells, eight strips) in proportion. After incubating for 30 min at room temperature (25 °C) under light-protected conditions, the absorbance was measured at a wavelength of 405 nm using a light-absorbing enzyme-labeled instrument (VersaMax, Beijing Yuechangxing Technology Co., Ltd., Beijing, China). Three repeated samples were measured for each treatment after calculating the SOD activity in the tissue samples of *I. indigotica*, using GraphPad.Prism 8 software was used for visualization. SOD is the first line of defense in the plant antioxidant system. It relies on metal cofactors (Cu^2+^/Zn^2+^, Mn^3+^, or Fe^3+^) to catalyze the dismutation reaction of superoxide anions (O^2−^). The reaction can be expressed as [59]:

$$2O_{2}^{\cdot -}+2H^{+}\;\to \;H_{2}O_{2}+O_{2}$$

Through this reaction, SOD rapidly converts highly toxic O^2−^ into H_2_O_2_ and releases O_2_, thereby effectively alleviating the oxidative stress caused by ROS in cells.

### 4.7. Effects of Alkaline Stress on the Accumulation of Indole Alkaloids in I. indigotica

Indole alkaloids are secondary metabolites produced during plant growth and have essential ecological functions in adapting to environmental stress. Indole alkaloids are mainly enriched in the leaves of *I. indigotica*, including indigo, indirubin, tryptanthrin, etc. [42]. According to the 2020 edition of *the Chinese pharmacopeia*, indigo and indirubin are the quality control components of *I. indigotica* Fort. Indigo and indirubin can be natural antioxidants in the food or pharmaceutical industry [60]. Changes in the content of indigo and indirubin in *I. indigotica* under alkaline stress treatments (0 h (CK), 24 h, 72 h, 120 h) were detected using HPLC. The chromatograph was a Thermo U3000 series, and the column was an Agilent C_18_ (250 mm × 4.6 mm, five μm particle size). Indigo and indirubin control products were purchased from Sichuan Vicki Biotechnology Co., Ltd. (Chengdu, China), and about 0.5 g of powdered *I. indigotica* leaves from the alkaline stress treatment groups were precisely weighed and dissolved in 10 mL of N, N-dimethylformamide solution. Vortex shaking for about 5 min, ultrasonic extraction for 45 min (power 300 W, frequency 80 Hz), cooled to room temperature, and N, N-dimethylformamide was added to the scale. After shaking well, the test solution was filtered through a 0.45 μm organic microporous filter membrane to determine the test solution. The elution procedure was based on a methanol (A)-ultrapure water (B) gradient as the mobile phase [61]. Isocratic elution (0–20 min, 75:25); Flow rate: 1.0 mL/min; Detection wavelength: 290 nm; Column temperature: 25 °C; Injection volume: 10 μL [62].

### 4.8. Yeast One-Hybrid Assay

Yeast One-Hybrid (Y1H) is a typical method for detecting protein–DNA interactions [63]. WRKY transcription factors (TFs) widely regulate plant biotic and abiotic stress responses, growth, and development [64,65]. WRKY transcription factors specifically bind to the cis-acting element W-box (TTGACT/C) in the promoter region of downstream target genes to activate or inhibit the expression of downstream target genes [66,67]. *AtWRKY45* plays a vital role in response to saline–alkaline and osmotic stress [68]. When constructing a phylogenetic tree, *IiWRKY54* with a strong genetic relationship with *AtWRKY45* was selected [69,70], which indicated that *IiWRKY54* may be involved in the regulation of saline–alkaline stress response of *I. indigotica*. According to qRT-PCR analysis, *IiSOD* genes with high expression under alkaline stress were screened, and the PlantCARE website screened *IiSOD2* and *IiSOD7* genes with W-box cis-acting elements. It was found that *IiSOD2* and *IiSOD7* have both W-box cis-acting elements and high expression under alkaline stress. Therefore, *IiSOD2*, *IiSOD7*, and *IiWRKY54* were selected for yeast experimental verification.

The recombinant plasmid was constructed using homologous recombination, and homology arm primers were designed using the Tiangen Seamless Cloning Primer Online Tool (Available online: http://yw.tiangen.com/) based on the CDS sequence of the *IiWRKY54* transcription factor and the pJG4-5 vector sequence 20 bp above and below the EcoRI restriction site. The W-box fragment sequences of the promoter regions of the two *IiSOD* genes and the pLacZi vector sequence of 20 bp up and down the EcoRI restriction site were used to design homologous arm primers. The primer sequences for this experiment are detailed in Supplementary Table S3. After obtaining the target gene, the recombinant vector was constructed by the constant temperature one-step method, and the two *IiSOD* target genes were ligated with the linearized pLacZi vector. The *IiWRKY54* gene was ligated to the linearized pJG4-5 vector (pLacZi and JG4-5 had Amp resistance from Coolaber, Beijing, China). The bait vector constructed by pLacZi-*IiSOD2* and pLacZi-*IiSOD7*, and the prey vector constructed by pJG4-5-*IiWRKY54* were co-transformed into the EGY48 yeast strain (purchased from Shanghai Weidi Company (Shanghai, China)). No-load pLacZi-no-load pJG4-5, no-load pJG4-5-pLacZ-*IiSOD2*, no-load pJG4-5-pLacZ-*IiSOD7*, and pJG4-5-*IiWRKY54*-no-load pLacZi were transformed into the EGY48 yeast strain as negative controls to exclude false negative results. The transformed yeast was plated on SD/-Trp-Ura-Broth medium and cultured at 30 °C for 3-5 days. The positive bacteria were selected and cultured in SD/-Trp-Ura-Broth medium, and the plate was coated with chromogenic medium and cultured at 30 °C for 1–3 days. The interaction results were determined by observing the color change in the colony.

### 4.9. Statistical Analysis

One-way ANOVA (Tukey’s test) was used to determine significant differences between the groups in qRT-PCR, superoxide dismutase activity, and indigo and indirubin content. Three replications were performed for both control and treatment groups.

## 5. Conclusions

In this study, the *SOD* gene family of *I. indigotica* was systematically analyzed, and 9 *IiSOD* members were identified. Based on the conserved domains, these genes were classified into three subfamilies: Cu/Zn-SOD, Fe-SOD, and Mn-SOD, which were unevenly distributed across seven chromosomes. Under alkaline stress, the SOD enzyme activity of *I. indigotica* was significantly increased, indicating that SOD plays a vital role in antioxidant defense. The combination of qRT-PCR and phylogenetic analyses revealed that members of the Fe-SOD and Mn-SOD subfamilies responded particularly well to alkaline stress. Alkaline stress showed significant and dynamic changes in the content of indigo and indirubin in leaves of *I. indigotica*, with the accumulation levels decreasing and increasing with stress duration.

The transcription factor *IiWRKY54* regulates the transcriptional activation of *IiSOD2* and *IiSOD7*; among them, the expression level of *IiSOD7* was significantly up-regulated under alkaline stress. In this study, the biological information of the *IiSOD* gene family was systematically identified, which laid a foundation for an in-depth analysis of the molecular mechanism of the response of the *IiSOD* gene to saline–alkaline conditions. Studies have shown that in addition to improving the stress resistance of plants, the regulation of SOD activity also plays a positive role in the active ingredients of plants. In the future, the use of homologous or heterologous expression systems combined with gene editing methods to further verify the function of these genes will provide direction for plant breeding and stress resistance.

## Figures and Tables

**Table 1:** Identification data of *IiSOD* genes in *I. indigotica* genome.

Gene Name	Gene ID	Amino Acids	Chromosome Location	pI	Molecular Weight (Da)	Subcellular Location	Pfam Domain
*IiSOD1*	Iin00748.t1	260	Chr1	7.02	29,539.48	mitochondrion	IMA, IMC
*IiSOD2*	Iin05388.t1	167	Chr2	6.48	17,057.09	chloroplast	CZ
*IiSOD3*	Iin06861.t1	307	Chr2	4.85	34,597.74	chloroplast	IMA, IMC
*IiSOD4*	Iin08997.t1	153	Chr3	5.45	15,163.77	chloroplast	CZ
*IiSOD5*	Iin09368.t1	323	Chr3	7.64	34,325.95	chloroplast	CZ
*IiSOD6*	Iin15648.t1	232	Chr4	8.74	25,463.07	mitochondrion	IMA, IMC
*IiSOD7*	Iin22527.t1	257	Chr6	7.74	28,582.51	chloroplast	IMA, IMC
*IiSOD8*	Iin24760.t1	182	Chr7	9.46	20,410.29	mitochondrion	IMA, IMC
*IiSOD9*	Iin27319.t1	187	Chr7	6.79	20,318.94	mitochondrion	IMA, IMC

pI: isoelectric points; CZ: Copper/zinc superoxide dismutase; IMA: Iron/manganese superoxide dismutases, alpha-hairpin domain; IMC: Iron/manganese superoxide dismutases, C-terminal domain.

## Data Availability

Data sharing not applicable to this article as no datasets were generated or analyzed during the current study.

## Supplementary Materials

The supporting information can be downloaded at: https://www.mdpi.com/article/10.3390/ijms26178131/s1.
