# Supplementary material for: Genome-Wide Expression Profile of SOD Gene Family in Isatis indigotica and the Key Role of IiSOD2 and IiSOD7 in Alkaline Stress
Source: Int J Mol Sci. 2025 Aug 22;26(17):8131. doi: 10.3390/ijms26178131 (PMC12427683; doi:10.3390/ijms26178131)
Supplement: Supplementary file 1 [file ijms-26-08131-s001.zip › ijms-3761503-supplementary.pdf]

Supplementary Material

SignalP-5.0 prediction (Eukarya): IiSOD1

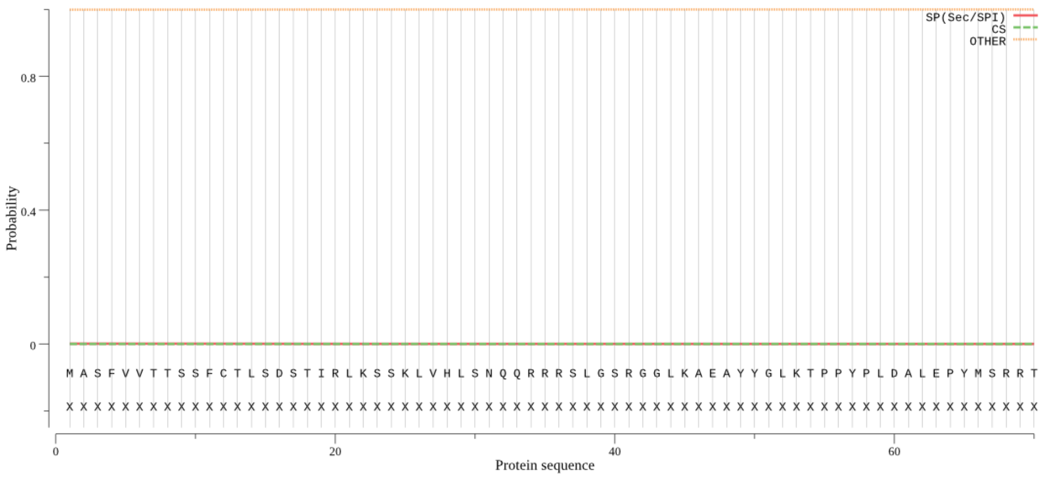

SignalP-5.0 prediction (Eukarya): IiSOD2

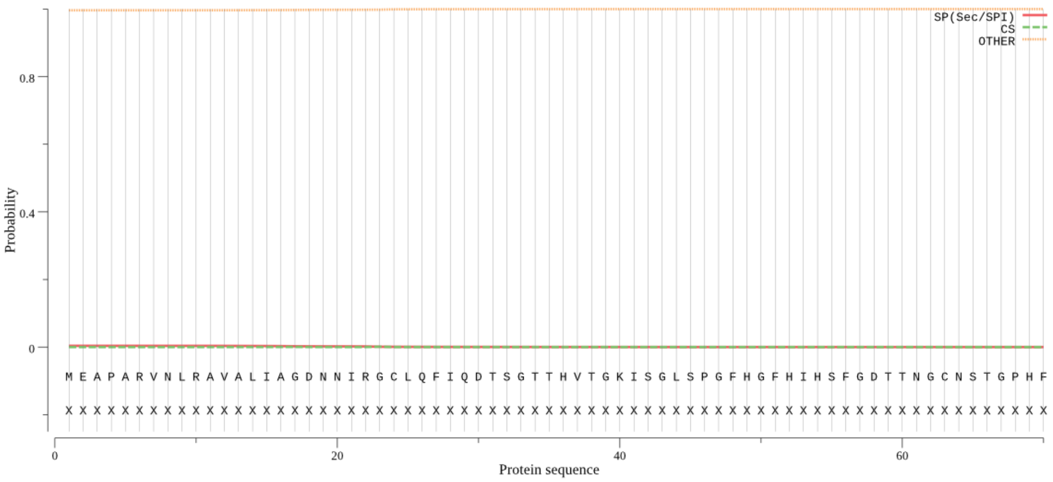

SignalP-5.0 prediction (Eukarya): IiSOD3

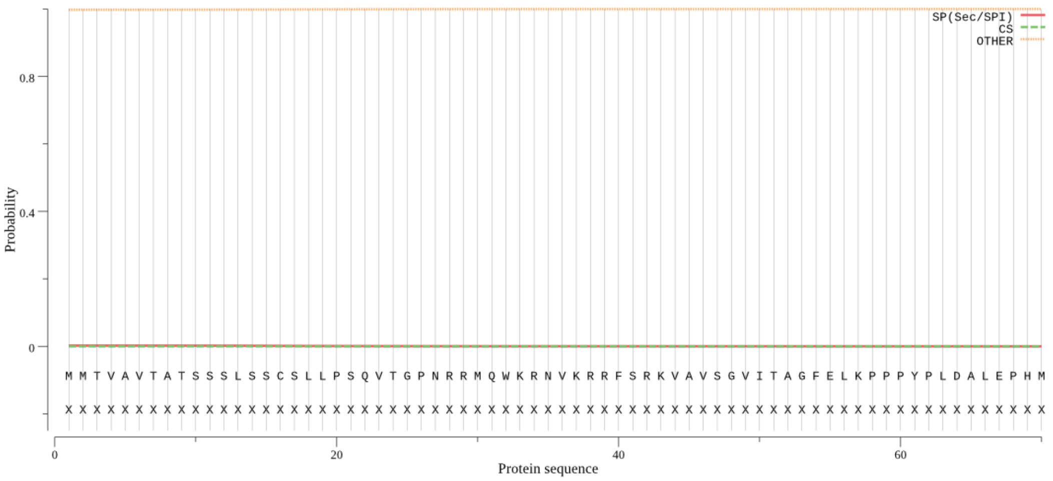

SignalP-5.0 prediction (Eukarya): IiSOD4

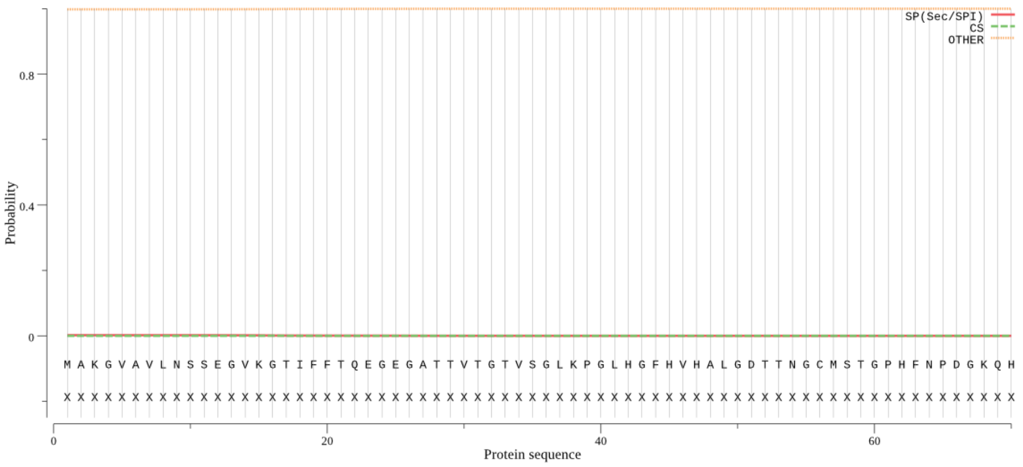

SignalP-5.0 prediction (Eukarya): IiSOD5

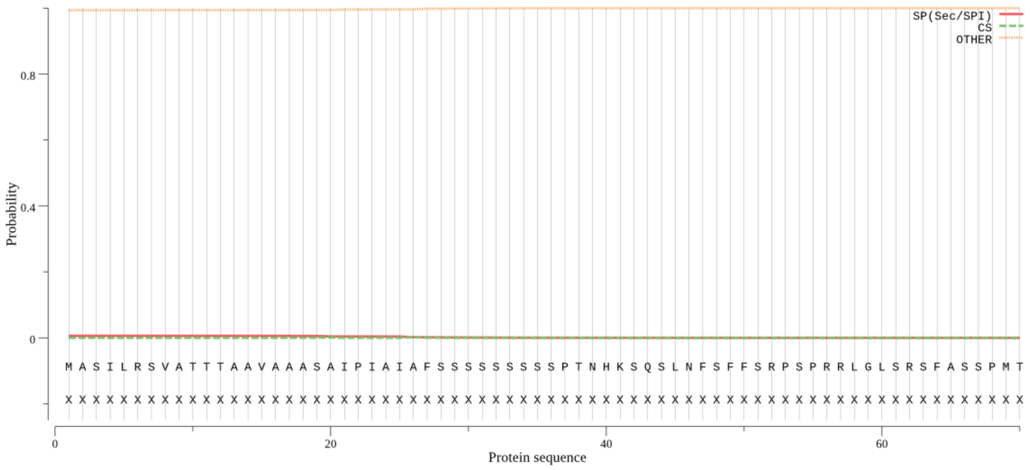

SignalP-5.0 prediction (Eukarya): IiSOD6

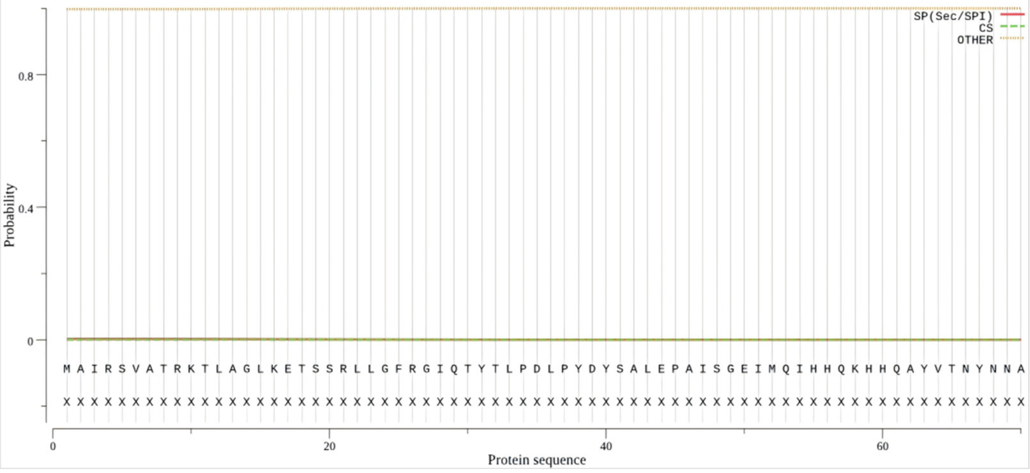

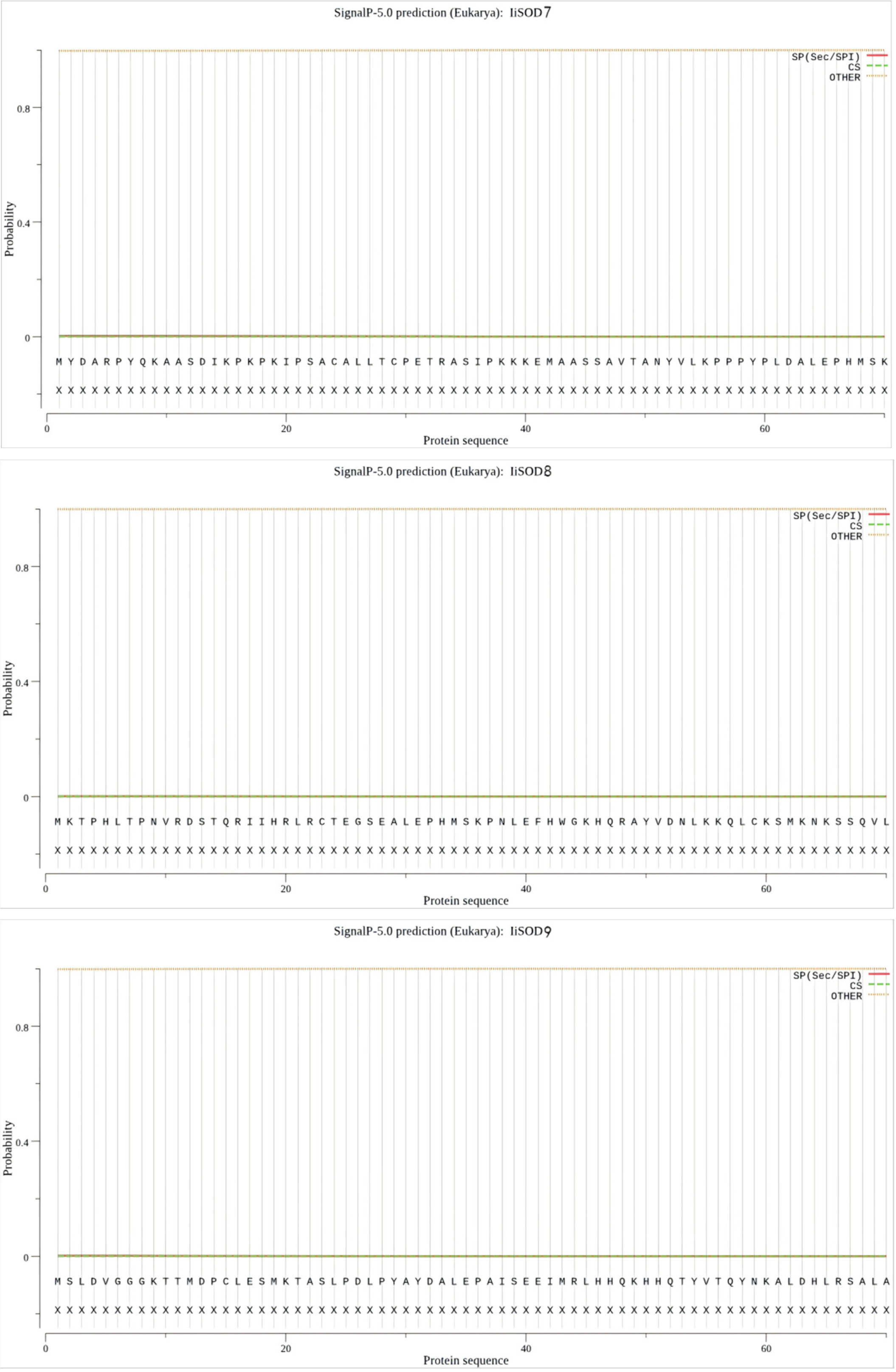

Supplementary Figure S1: Signalling peptides of IiSOD proteins.

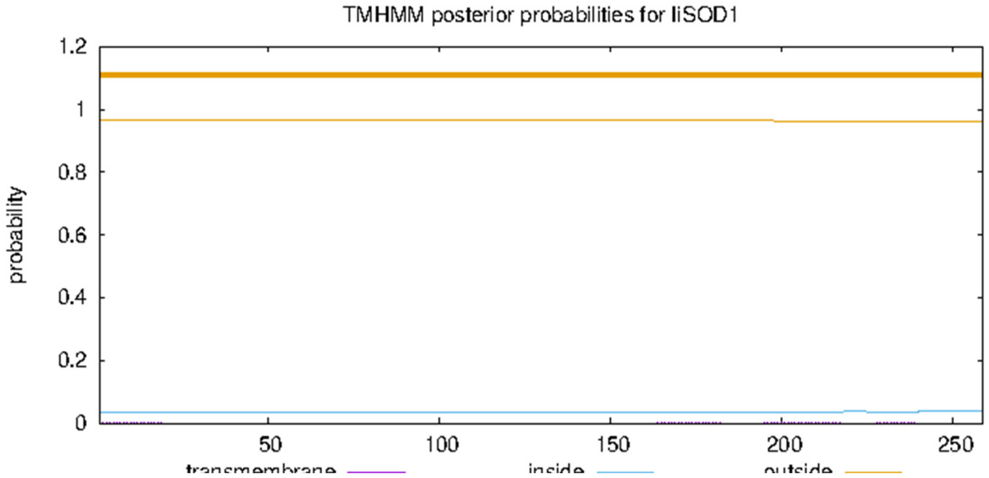
TMHMM posterior probabilities for liSOD1
probability
1.2
1
0.8
0.6
0.4
0.2
0
50
100
150
200
250

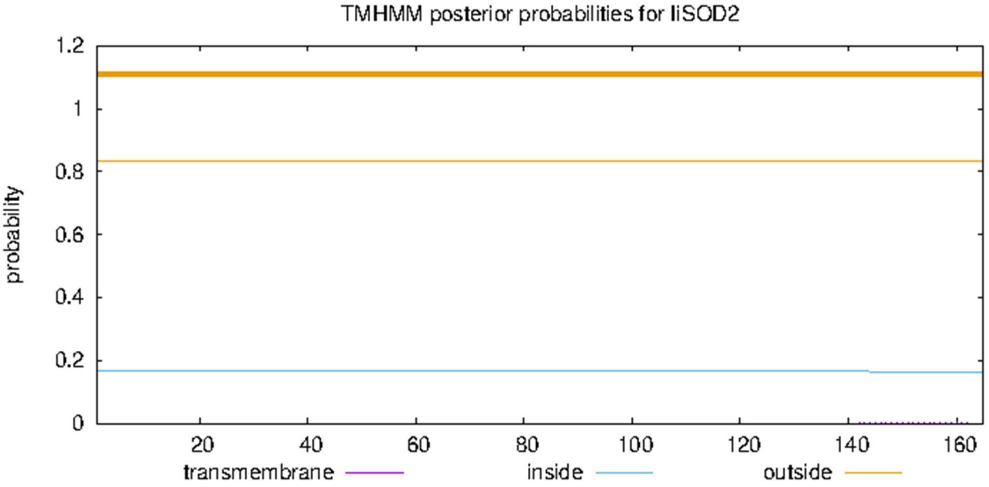
TMHMM posterior probabilities for liSOD2
probability
1.2
1
0.8
0.6
0.4
0.2
0
20
40
60
80
100
120
140
160
transmembrane
inside
outside

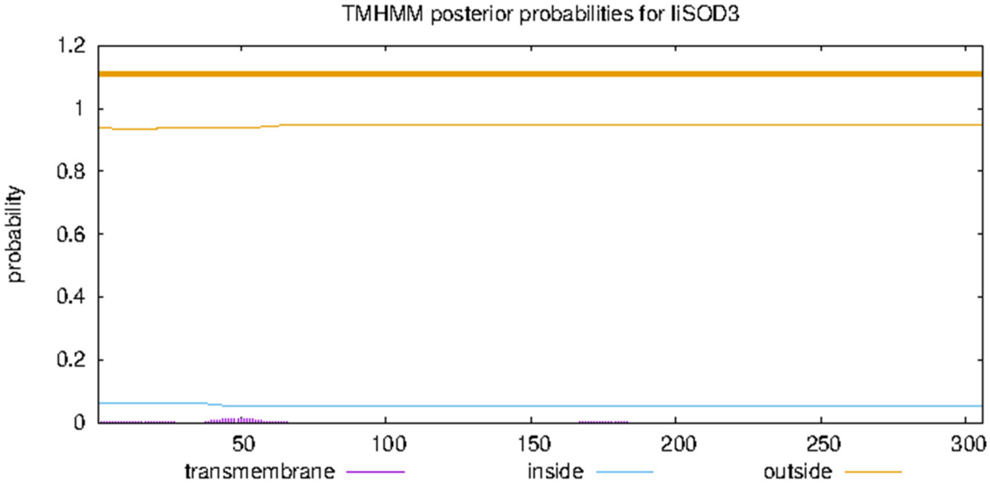
TMHMM posterior probabilities for liSOD3
probability
1.2
1
0.8
0.6
0.4
0.2
0
50
100
150
200
250
300
transmembrane
inside
outside

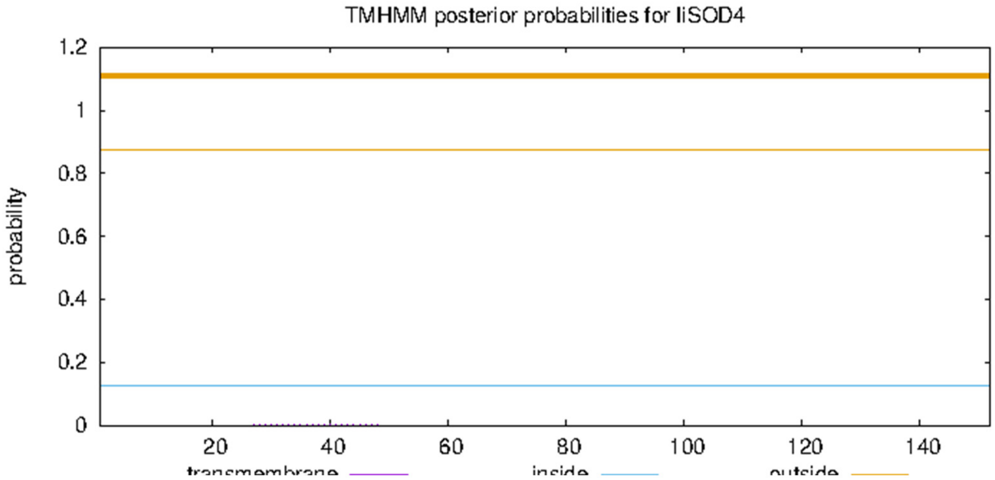
TMHMM posterior probabilities for liSOD4
probability
1.2
1
0.8
0.6
0.4
0.2
0
20
40
60
80
100
120
140
transmembrane
inside
outside

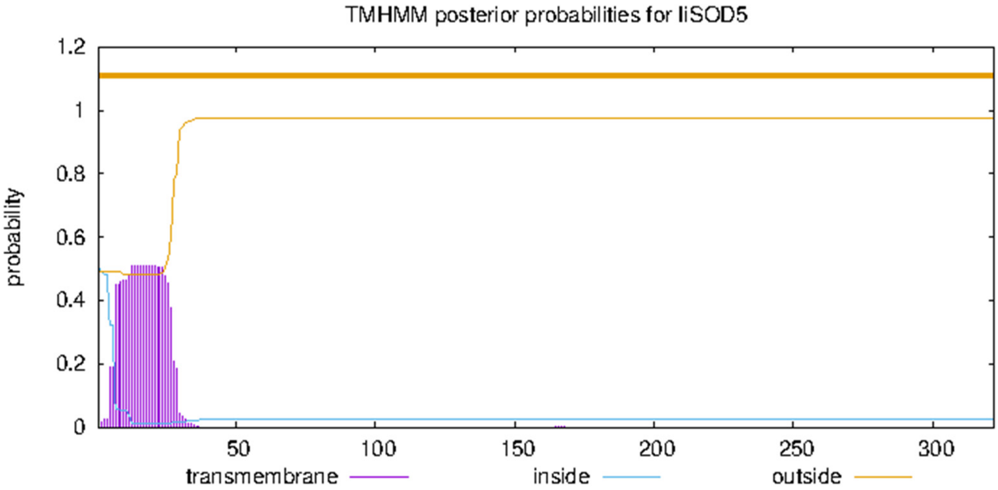
TMHMM posterior probabilities for liSOD5
probability
1.2
1
0.8
0.6
0.4
0.2
0
50
100
150
200
250
300
transmembrane
inside
outside

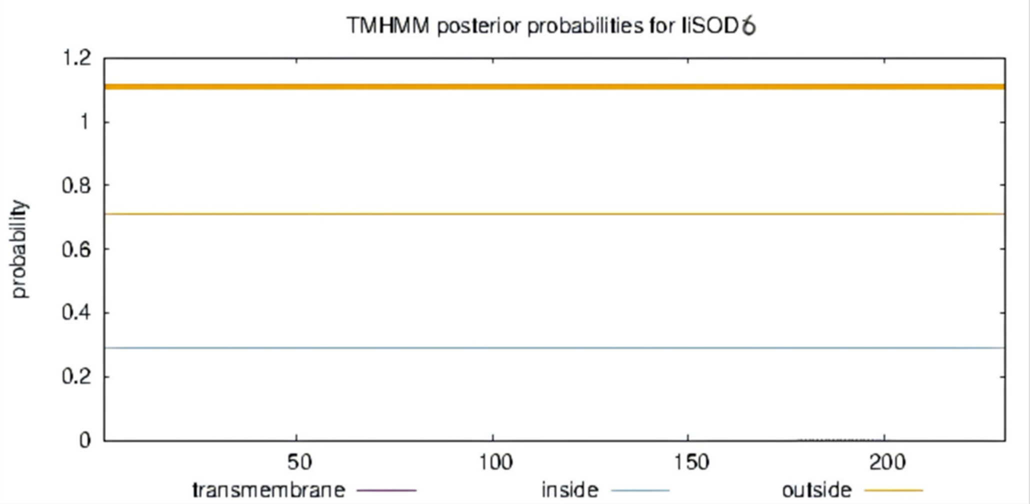
TMHMM posterior probabilities for liSOD6
probability
1.2
1
0.8
0.6
0.4
0.2
0
50
100
150
200
transmembrane
inside
outside

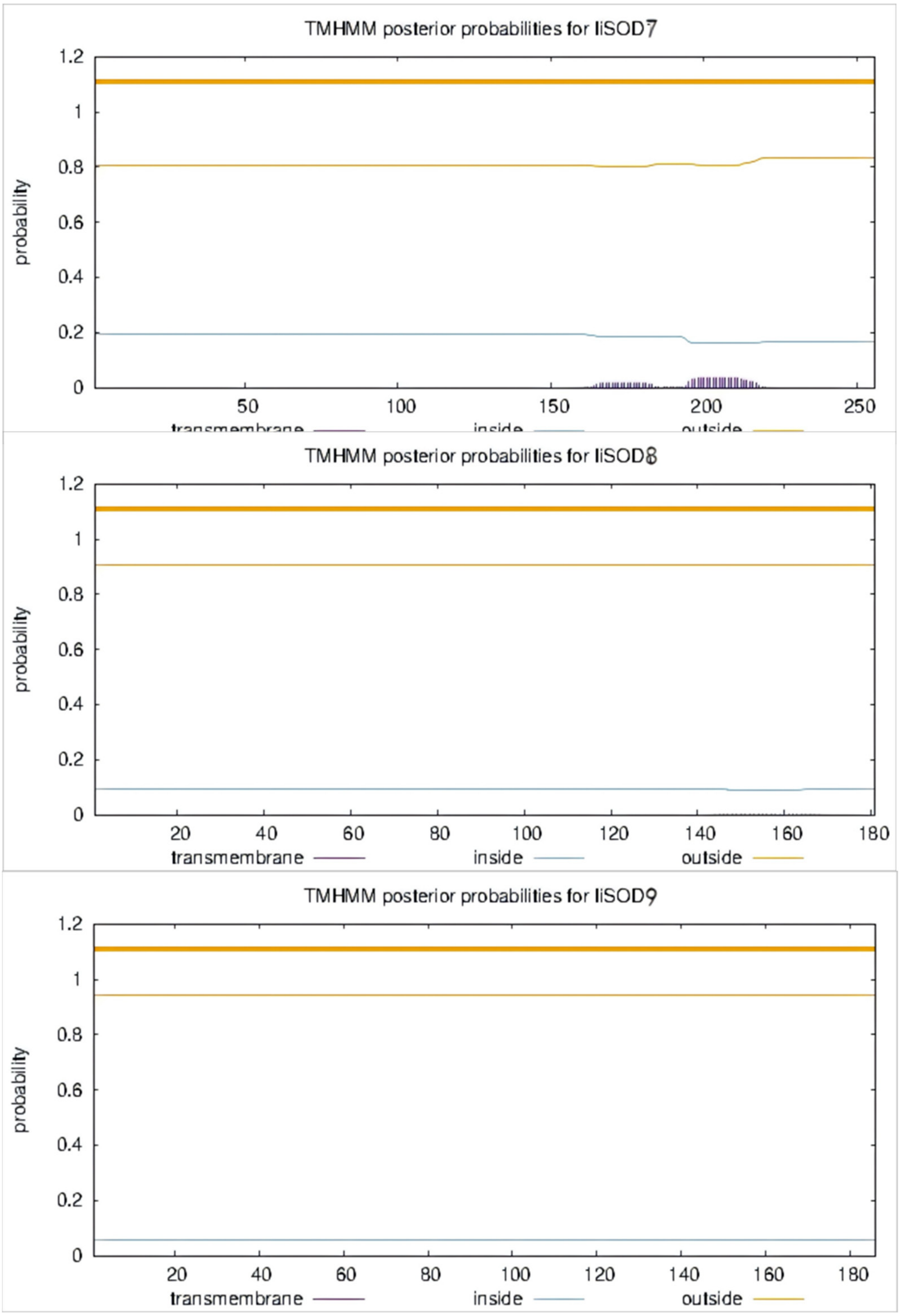

Supplementary Figure S2. Transmembrane domains of IiSOD proteins.

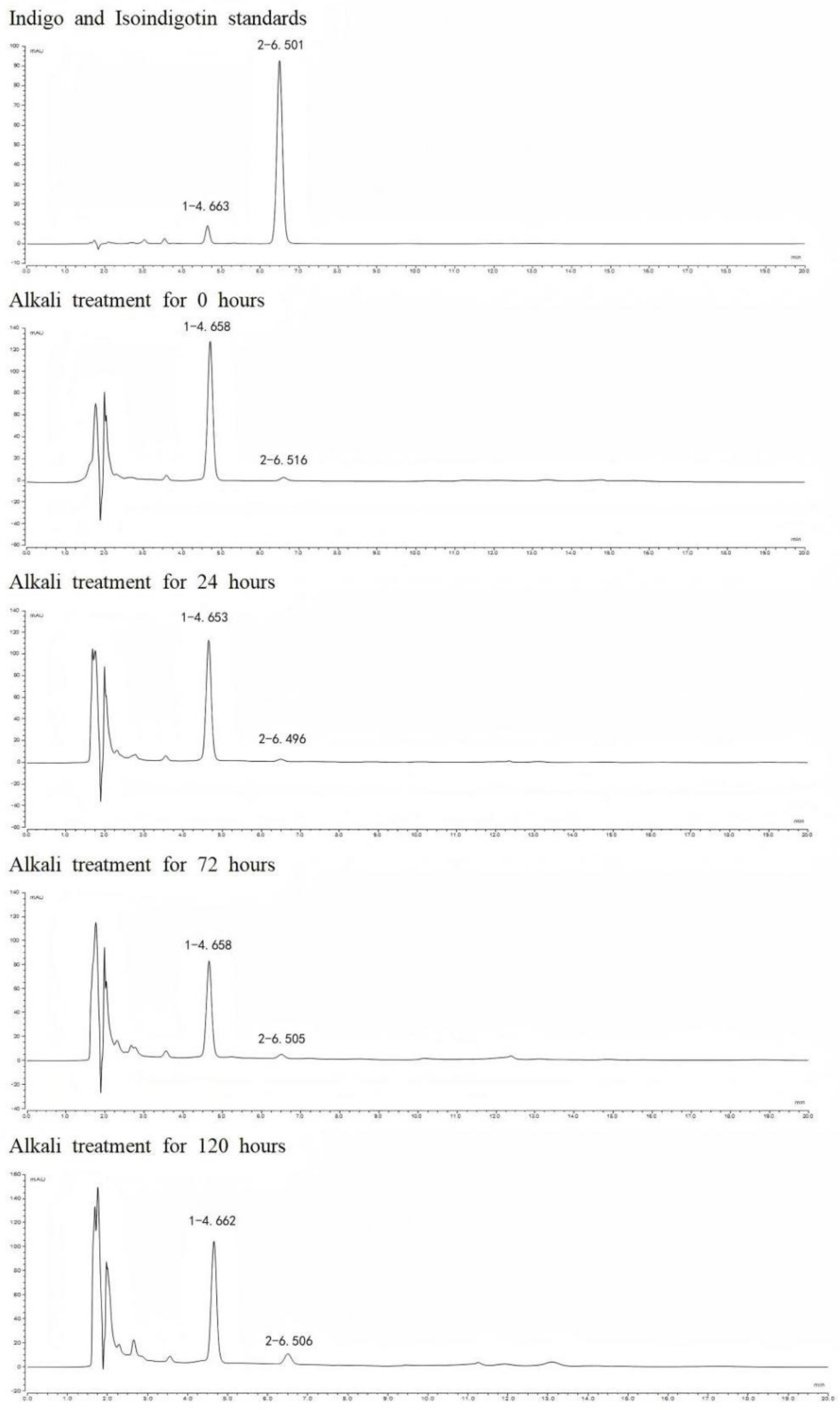

Supplementary Figure S3. High Performance Liquid Chromatography. the peak time and peak area of 1- indigo and 2-indirubin.
